# New Drugs, Appliances, and Things Medical

**Published:** 1892-12-17

**Authors:** 


### NEW DRUGS, APPLIANCES, AND THINGS MEDICAL.

[All preparations, appliances, novelties, etc., of which a notice is desired, should be sent for the Editor, to care of The Manager, 140, Strand, London, W.C.]

### ELECTRO-MEDICAL INSTRUMENTS (SCHALL).

We have received from Mr. Schall, of 55, Wigmore Street; W., his illustrated price list of electro-medical instruments. This has a useful and interesting introduction on the management and construction of these instruments. In it is given a concise and intelligent *résumé* of the laws of electricity, and thus the reader is enabled to get at the points which are most useful in the preservation and use of the electrical apparatus he may be using. The illustrated list shows examples of all the best known forms of electrical apparatus, and when we find that the greatest experts and many hospitals, where electrical treatment is an important feature of the therapeutical appliances employed, make use of Mr. Schall's skill, we need not state that the workmanship is all that can be desired. We note particularly the forms of battery on pp. 64 and 88. Of specimens of these we can speak from actual experience with the highest praise. Besides the usual electrical apparatus, the catalogue shows how the force applied for electrical lighting can be transformed into that for electrical treatment, and the necessary instruments are fully illustrated. The latest improvements in the various "scopes" for examining the cavities of the body receive due explanation and illustration, so that, in conclusion, we may say that perusal of the catalogue enables the reader to get a very good idea of the present state of electrical therapeutics.

### EUCALYPTUS OIL.

Platypus Brand.

We have received a sample of "The Tasmanian Eucalyptus Oil Company's" pure Oleum Eucalypti globuli for notice. Our examination of the specimen bears out the analysis which the pamphlet issued by the company contains. The oil has a remarkably high eucalyptol percentage, is clean, volatile, and fragrant. We have repeatedly noticed the methods of employment of eucalyptus oil in our columns, being among the first to fully illustrate the views of Mr. Curgenven on its use in scarlet fever and other infectious diseases. As there has been a large quantity of very inferior eucalyptus oil in the market owing to the demand for the oil during the influenza epidemic having used up the best specimens, the profession may be glad to know of such a good brand as the above. It can be obtained direct from 138, Leadenhall Street.

